# Correlations Between Inflammatory Biomarkers and COPD Severity in a Real-Life Setting

**DOI:** 10.3390/jcm14186481

**Published:** 2025-09-15

**Authors:** Elena-Andreea Moaleș, Lucia Corina Dima-Cozma, Cristina Andreea Adam, Andrei Mihordea, Doina-Clementina Cojocaru, Paraschiva Postolache, Maura Gabriela Felea, Robert Negru, Ioana Mădălina Zota, Mitică Ciorpac, Romică Sebastian Cozma, Florin Dumitru Petrariu, Maria Magdalena Leon, Florin Mitu

**Affiliations:** 1 Department of Medical Specialities I, Faculty of Medicine, “Grigore T. Popa” University of Medicine and Pharmacy, University Street No. 16, 700115 Iași, Romania; 2 Elytis Hospital Iasi, Gheorghe Săulescu Street No. 43 A, 700010 Iasi, Romania; 3 Clinical Rehabilitation Hospital, Pantelimon Halipa Street No. 14, 700661 Iași, Romania; 4 Advanced Research and Development Center for Experimental Medicine “Prof. Ostin C. Mungiu”—CEMEX, “Grigore T. Popa” University of Medicine and Pharmacy, 700115 Iași, Romania; 5 2nd Surgery Department, Faculty of Medicine, “Grigore T. Popa” University of Medicine and Pharmacy, University Street No 16, 700115 Iași, Romania; 6 Department of Preventive Medicine and Interdisciplinarity, Faculty of Medicine, “Grigore T. Popa” University of Medicine and Pharmacy, University Street No. 16, 700115 Iași, Romania; 7 Romanian Academy of Scientists, 050045 Bucharest, Romania; 8 Romanian Academy of Medical Sciences, 030167 Bucharest, Romania

**Keywords:** chronic obstructive pulmonary disease (COPD), inflammation, neutrophil-to-lymphocyte ratio (NLR), lymphocyte/HDL-C ratio (LHR), platelet-to-lymphocyte ratio (PLR), interleukin 8 (IL-8), interleukin 6 (IL-6), interleukin 1-beta (IL-1beta), tumor necrosis factor alpha (TNF-alpha)

## Abstract

**Background:** With a steady increase in prevalence, chronic obstructive pulmonary disease (COPD) is recognized as a leading contributor to global mortality, which underscores the need to identify prognostic biomarkers that are easy to use in everyday practice. As both local and systemic inflammation play a pivotal part in the development and progression of COPD, the purpose of our study was to assess the role of inflammatory markers in estimating disease severity (GOLD 1 to 4) to guide the management and treatment of patients with COPD, thus helping to reduce episodes of decompensation and fatal outcomes in both immediate and long-term contexts. **Methods:** We performed a descriptive observational analysis with a cross-sectional design including 100 patients with stable COPD. All subjects underwent standard clinical examination, lung function tests and a comprehensive inflammation blood panel. **Results:** We included 55 patients with mild–moderate COPD (GOLD 1–2), 33 patients with advanced stages of COPD (GOLD 3) and 12 patients with very severe forms of the disease (GOLD 4). Male sex, smoking status, BMI, IL-1 beta, NLR, and nocturnal heart rate were parameters with significant value for assessing COPD severity (*p* < 0.05 for all). NLR correlates significantly with the predicted distance in the 6 MWT (*p* = 0.039). Significant associations were identified between IL-8 and duration of exposure of biomass fuel (*p* = 0.045) as well as between IL-6 and pack-years (*p* = 0.024), with both prognostic and therapeutic impact. Furthermore, the analysis of the total cohort revealed positive correlations between serum IL-6 and FVC (%) (*p* = 0.033) or NLR and the ratio between FEV1 and FVC (*p* = 0.048). The neutrophil-to-lymphocyte ratio (NLR) proved to be a reliable indicator of stages 3–4 GOLD (AUC 0.66, threshold value 2.3045, *p* = 0.004—a one-point increase in NLR corresponded to a 2.148 increase in FEV1). **Conclusions:** NLR emerged as a simple, accessible and highly informative biomarker in COPD, linked to disease severity and functional decline. Its integration into routine assessment could enhance prognosis and guide clinical decisions, a finding that warrants confirmation in future multicenter studies.

## 1. Introduction

Chronic obstructive pulmonary disease (COPD) is currently considered one of the main global causes of death, placing an immense economic burden on worldwide healthcare systems [1]. COPD is a chronic, multifaceted respiratory disorder marked by ongoing and often irreversible airflow limitation [2,3]. COPD patients present chronic respiratory symptoms and are at risk for multiple acute exacerbations, which accelerate disease progression [4]. Exposure to tobacco smoke, household gases and outdoor air pollution cause chronic airway inflammation, which is responsible for airway and alveolar space remodeling [5]. Airway inflammation, observed in COPD physiopathology, includes recruitment of neutrophils, macrophages and cytotoxic T lymphocytes (CD8+) [6].

Oxidative stress plays a major role in the onset and evolution of COPD, with recent research focusing on the post-translational modification of proteins (carboxylation and citrullination), which generates self-antigens and promotes an auto-immune response [7,8,9]. Neutrophils are recognized as key cellular elements in the inflammatory mechanisms of COPD and have been linked to the intensity of airflow obstruction. [10]. They are abundantly present in the airway secretions of individuals with COPD, releasing a variety of pro-inflammatory mediators and enzymes (matrix metallo-proteinases and neutrophil elastase (NE)), which promote airway remodeling and emphysema [11,12]. NE impairs macrophage function and promotes persistent airway inflammation by stimulating the production of macrophage-derived extracellular traps (METs), which contain extracellular DNA and oxidative enzymes, which exacerbate chronic pulmonary inflammation and contribute to disease progression in COPD [13].

The neutrophil-to-lymphocyte ratio (NLR), an indicator of immune cell imbalance that can be derived from a standard peripheral blood test, has recently emerged as a diagnostic and prognostic maker in various disease [14]. Among individuals diagnosed with COPD, NLR shows significant association with disease severity, frequency of acute flare-ups, likelihood of hospitalization, and the need for assisted ventilation or mortality risk [15].

There is increasing interest in how inflammation contributes to the pathophysiology and clinical progression of COPD. In addition to local, mucosal inflammation, patients with stable COPD often display persistent low-grade systemic inflammation, as evidenced by the elevated serum concentrations of inflammatory biomarkers [16]. These include C-reactive protein (CRP), interleukin-1 beta (IL-1 beta), interleukin-6 (IL-6), interleukin-8 (IL-8) and tumor necrosis factor-alpha (TNF-alpha), which are among the most extensively evaluated inflammatory markers in COPD research and exhibit increased levels when compared with healthy subjects [17,18]. Circulating neutrophils and thrombocytes appear to have a pro-thrombotic-pro-inflammatory phenotype in COPD patients. The platelet-to-lymphocyte ratio (PLR) and the neutrophil-to-lymphocyte ratio (NLR) reflect distinct immune-inflammatory mechanisms and can be readily measured using routine hematological analysis. They have documented prognostic implications in cardiovascular [19,20] and autoimmune disease [20], as well as in malignancies [21,22] and COPD [23].

As such, systemic inflammation is an important hallmark even in stable COPD. We hypothesized that using circulating inflammatory markers might assist in staging COPD severity according to GOLD criteria.

## 2. Materials and Methods

### 2.1. Study Design

We conducted a prospective observational study of 100 patients diagnosed with clinically stable COPD, under optimal guideline-directed therapy, who were evaluated at the Clinical Rehabilitation Hospital in Iași from January to December 2022. We excluded patients aged under 35 years, those with persistent inflammatory disorders, neoplastic conditions, chronic alcohol use, low platelet counts or any acute medical event in the preceding 30 days (including acute COPD exacerbation) or any other severe chronic comorbidities that could impact respiratory symptoms or complete blood count (CBC) values. In addition, patients with a prior diagnosis of bronchial asthma or with clinical features suggestive of asthma–COPD overlap (ACO) were excluded from this study.

### 2.2. COPD Diagnosis

COPD diagnosis and severity (GOLD stages) were established based on post-bronchodilator forced expiratory volume in one second (FEV1), measured by standard spirometry, according to current guidelines [3]. The GOLD classification defines COPD severity as follows: stage 1 (mild), FEV1 ≥ 80%; stage 2 (moderate), FEV1 = 50–79%; stage 3 (severe), FEV1 = 30–49%; and stage 4 (very severe), FEV1 < 30%.

#### Pulmonary Function

All of the participants in this study underwent spirometry to evaluate lung function. An expert examiner performed the measurements, and each participant had at least three accurate measures, in accordance with current professional society standards. Key spirometry metrics included the maximum expiratory flow when 50% of the FVC was exhaled [MEF50], FVC, the FEV1/FVC ratio and post-bronchodilatator FEV1. To determine the expected values for all spirometry outcomes, the following data were recorded: core body temperature, atmospheric pressure, humidity level [BTPS conditions] and the patients’ demographic data, including birth sex, ethnicity, age, height and weight.

Current guidelines [3,24] state that an FEV1/FVC ratio greater than 70%, along with FEV1, FVC, and MEF50 values above 80% of their predicted thresholds were considered normal values. Airflow limitation was defined by the Global Initiative for Chronic Obstructive Lung Disease (GOLD) as an FEV1/FVC ratio below 70%. The severity of COPD was classified into four stages: Stage 1 (FEV1 ≥ 80%), stage 2 (FEV1 = 50–79%), stage 3 (FEV1 = 30–49%) and stage 4 (FEV1 < 30%).

### 2.3. Measurements

All patients underwent a complete anamnesis and physical evaluation, conducted by a certified medical provider. According to the smoking status, patients were divided into never, past and current smokers. The degree of breathlessness was evaluated using the modified medical research council (mMRC) dyspnea scale [25].

The 6 MWT was conducted following American Thoracic Society recommendations [26] upon hospital admission. The test is a self-paced protocol that allows the patient to choose their walking pace and stop and rest as needed during the test. We recorded the 6 min walk distance (6 MWD) as the total number of meters covered by each patient.

In accordance with institutional protocol, blood was drawn in a fasting state (after 12 h without food) in the morning upon hospital admission. Samples were analyzed in the laboratory of the Clinical Rehabilitation Hospital using the Pentra DF Nexus Hematology System^®^ (Horiba Healthcare, Kyoto, Japan) for CBC and Transasia XL 1000 Fully Automated Biochemistry Analyzer (Transasia Bio-Medicals Ltd., Mumbai, India) for biochemical investigations.

We evaluated the following inflammatory parameters: CRP, IL-1b, IL-6, IL-8 and TNF-alpha. The NLR was calculated using the standard formula: NLR = N/L, where N and L represent the absolute neutrophil (N) and lymphocyte (L) counts from the CBC. PLR was calculated according to the following formula: PLR = P/L, using the absolute platelets (P) and lymphocyte (L) counts obtained from the CBC. The LHR was calculated according to the following formula: LHR = L/HDL, where L represents the absolute lymphocyte (L) count determined from the CBC, and HDL-C is expressed in mg/dl.

Anthropometric evaluations were performed in triplicate. Body weight and height were measured in the morning on the day of admission, while patients were lightly dressed and barefoot. The body mass index (BMI) was calculated using the following formula: weight (kg) divided by height (m^2^). Waist circumference (WC) was measured with the tape placed horizontally at the level of the right iliac crest, following a normal exhalation.

#### 2.3.1. Laboratory Data

Both during enrollment and six months after the initial assessment, biological samples were taken. The pro-inflammatory status was investigated using the following parameters: complete blood count, lipid panel (including low-density lipoprotein cholesterol (LDL-C), HDL-C and triglycerides), fasting serum glucose and a set of inflammation-related biomarkers, including interleukin (IL)-8, IL-6, IL-1 beta (IL-1β) and tumor necrosis factor (TNF)-alpha. The analyses were carried out at the Advanced Research and Development Center for Experimental Medicine “Prof. Ostin C. Mungiu”—CEMEX, Iasi, Romania, were the levels of IL-8, IL-6, IL-1β and TNF-alpha were measured from serum samples using standardized ELISA kits, according to the manufacturer’s protocols. The reference thresholds were IL-6 < 7 pg/mL, IL-8 < 15 pg/mL, IL-1β < 5 pg/mL and TNF-alpha < 8.1 pg/mL. All data were expressed in accordance with the International System of Units.

#### 2.3.2. The Six-Minute Walking Assessment (6 MWT)

The six-minute walking assessment (6 MWT) was conducted following ATS/ERS [27,28]. All enrolled participants performed the 6-min walk test (6 MWT) protocol to evaluate their functional exercise capacity. Prior to the test, anthropometric measurements (weight, waist circumference), and previously taken medication (kind and dosage) were made. A 30 m long corridor was used, and the number of completed laps (both full and partial) was documented. Additional parameters, such as test duration, arterial pressure, perceived dyspnea (rated using the Borg scale) and peripheral oxygen saturation were collected both before and after the walking session. The most frequently reported symptoms during testing were dizziness; chest discomfort, suggestive of angina; and lower limb pain affecting the legs, calves, or hips.

#### 2.3.3. Multidisciplinary Management

The patients enrolled in this study benefited from integrative, multidisciplinary care that addressed modifiable cardiovascular risk factors such as altered lipid metabolism and impaired glucose regulation, following the ESC guidelines [29]. The interventions included blood pressure control, weight control optimization, dietary counseling, psychotherapy, quitting smoking and physical activity.

### 2.4. Statistical Analysis

Statistical evaluation was conducted using SPSS software, version 26.0 (IBM Corp., Chicago, IL, USA). For continuous variables, the assumption of normality was verified using the Shapiro–Wilk test. Data were reported as mean ± standard deviation (SD) for the variables showing a Gaussian distribution. Independent samples *t*-tests and ANOVA were applied to compare normally distributed continuous data. The Mann–Whitney and Kruskal–Wallis tests were applied to compare continuous data that were not normally distributed, while the Pearson’s chi-squared test with the Fisher correction was used for categorical variables. The receiver operating characteristic (ROC) analysis was performed in order to evaluate the NLR, PLR and LHR potential to discriminate between the GOLD stages 3–4 and 1–2 and to identify the corresponding cutoff values, calculated using the Youden index, which maximized the balance between sensitivity and specificity. The strength and direction of statistically meaningful associations identified during this study were assessed via Pearson’s or Spearman’s correlation coefficients, depending on the data distribution. A *p*-value < 0.05 was considered statistically significant and a *p*-value < 0.01 was considered statistically highly significant. Furthermore, regression modeling was employed to investigate the predictive value of inflammatory biomarkers in relation to COPD severity.

In addition, we performed calculations regarding the minimal required size of the sample using GPower 3.1.9.7 software, based on the statistical analysis conducted in our study. We found that, in order to perform chi-squared tests with a maximum of 6 degrees of freedom, assuming a large effect size (0.5), a standard alpha level (type I error) of 0.05 and a power of 0.8, the minimum required sample size is 55 subjects. In addition, in order to perform ANOVA-based comparisons among 3 samples assuming a large effect size (0.4), a standard alpha level (type I error) of 0.05 and a power of 0.8, the minimum required sample size is 66 subjects. It follows that our sample size of 100 patients is statistically sufficient for the purposes of this study.

### 2.5. Ethics Statement

All participants provided written informed consent prior to enrollment in this study. The research was conducted in compliance with the principles set forth in the Declaration of Helsinki [30] and received ethical clearance from the Ethics Committee of the University of Medicine and Pharmacy “Grigore T. Popa”, Iași (approval number 1183/17 January 2018).

## 3. Results

We included 100 subjects with a stable COPD, aged 67.02 years ± 9.303, divided into three groups as follows: 55 patients with mild–moderate COPD (GOLD 1–2), 33 patients with severe COPD (GOLD 3) and 12 patents with very severe COPD (GOLD 4). A significant proportion of patients (26%) were nonsmokers, with exposure to biomass fuel or occupational pollutants as major risk factors. Table 1 compares the demographic, anthropometric and inflammatory biomarkers assessed across the three analyzed subgroups. Table 2 presents the correlations between 6 MWT parameters and inflammatory biomarkers in our cohort.

**Figure 1:**
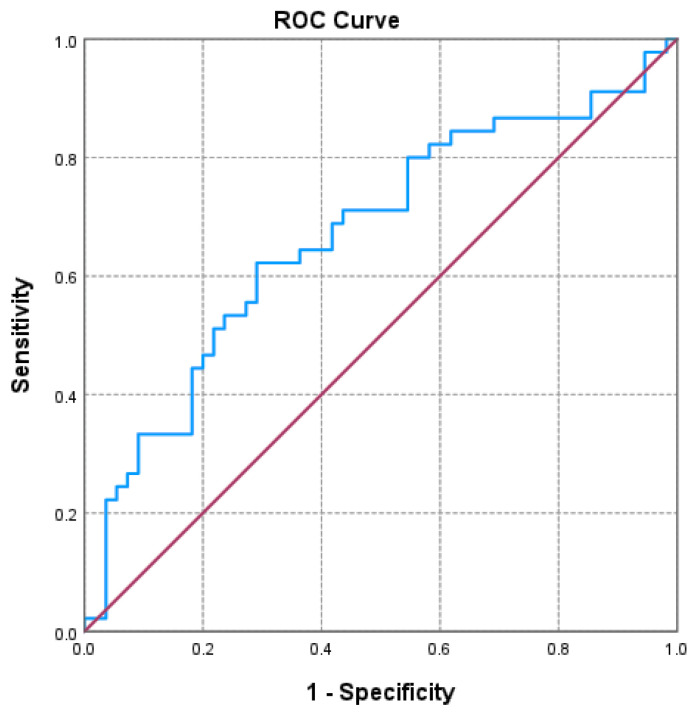
Receiver operating characteristic (ROC) curve evaluating the discriminatory performance of the neutrophil-to-lymphocyte ratio (NLR) in predicting GOLD stage 3–4 in COPD patients. The area under the curve (AUC = 0.669; *p* = 0.004) indicates a moderate discriminatory capacity.

**Figure 2:**
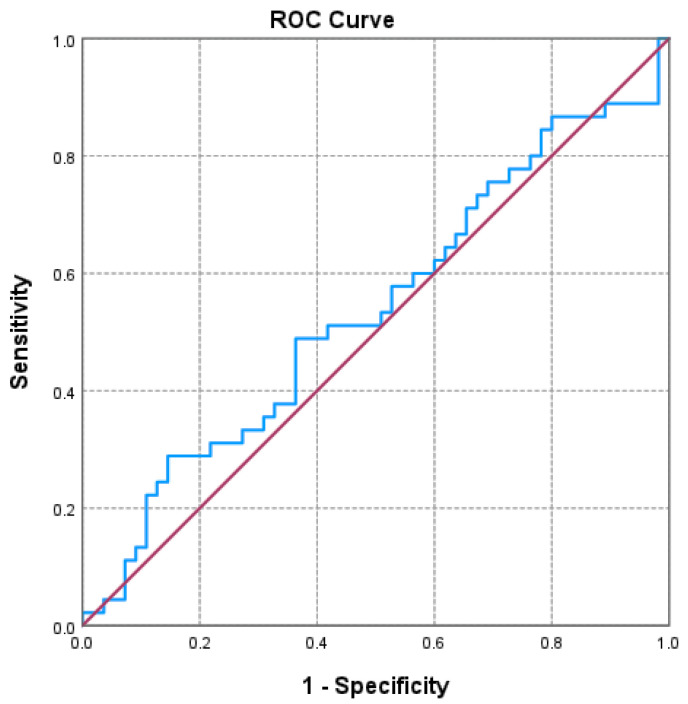
Receiver operating characteristic (ROC) curve evaluating the discriminatory performance of the platelet-to-lymphocyte ratio (PLR) in predicting GOLD stage 3–4 in COPD patients. The area under the curve (AUC = 0.536; *p* = 0.535) indicates a limited discriminatory capacity in this cohort.

**Figure 3:**
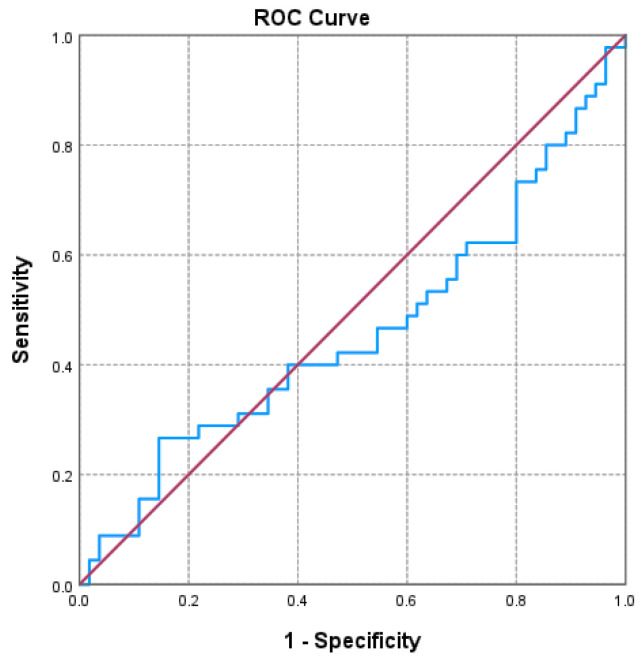
Receiver operating characteristic (ROC) curve evaluating the discriminatory performance of the lymphocyte-to-HDL-C ratio (LHR) in predicting GOLD stage 3–4 in COPD patients. The area under the curve (AUC = 0.459; *p* = 0.486) indicates a limited discriminatory capacity in this cohort.

Of the three markers investigated (Figure 1, Figure 2 and Figure 3 and Table 3), only the NLR varied statistically significantly with the GOLD stage, so we performed a ROC analysis to identify a possible cutoff value for it. We grouped the GOLD stage into two categories, forming two distinct subgroups (GOLD stages 1–2 versus GOLD stages 3–4). The receiver operating characteristic (ROC) analysis revealed a statistically significant area under the curve of 0.669, which means that using the NLR values as a predictor of GOLD stage is more efficient than relying on clinical stages. The cutoff value with the best values for sensitivity and specificity is NLR = 2.3045, for which the associated sensitivity is 62.2% and specificity is 70.9% (*p* = 0.004).

In the analyzed group, we observed statistically relevant associations between circulating IL-8 concentrations and the cumulative exposure to biomass combustion *(p* = 0.045), as well as between the IL-6 levels and smoking history, expressed in pack-years (*p* = 0.024) (Table 4).

The statistical analysis also revealed positive correlations between functional respiratory parameters and inflammatory markers such as the directly proportional relationship between serum IL-6 and FVC (%) (*p* = 0.033) or the NLR and the ratio between FEV and FVC (*p* = 0.048) (Table 5).

We performed multivariate analysis to predict COPD severity based on FEV1 as the dependent variable and inflammatory biomarkers as predictors. In this case, a multiple linear regression approach was deemed suitable, since both the dependent and independent variables are quantitative. We applied such a model in a first step but found multicollinearity between the variables LHR and IL-6, which were removed from the model. The model obtained is not very accurate (it explains 13.1% of the variation in the dependent variable, VEMS) and is not statistically significant. The remaining predictor variables contributed minimally to the model (the Beta coefficients are small). Table 6 shows the amount by which the value of the dependent variable (FEV1) changes when the independent variable is increased by one unit, e.g., a one unit increase in the NLR leads to a 2.148 increase in FEV1 (Table 6). In the group of 100 patients, the statistical relationship between the NLR and oxygen saturation was at the upper limit of statistical significance (*p* = 0.051). In addition, we observed a positive association between erythrocyte sedimentation rate and oxygen desaturation index, which reached statistical significance (*p* = 0.032) (Table 7).

## 4. Discussion

Inflammation has an essential role in COPD physiopathology, as demonstrated by the multiple correlations between systemic inflammatory markers and key functional parameters. We observed negative correlations between IL-6 and FVC% (r= 0.238, *p* = 0.033), and between NLR and the FEV1/ FVC ratio (r = −0.199, *p* = 0.048), suggesting an impact of inflammatory status on both lung volumes and airway obstruction. While NLR also showed a borderline significant correlation with the lowest nocturnal oxygen saturation values (r= −0.225, *p* = 0.051), the ESR was correlated with the nocturnal desaturation index (r = 0.246, *p* = 0.032), linking systemic inflammation to impaired gas exchange during sleep.

Pascual-González et al. highlighted a moderate correlation between the NLR and key COPD parameters, including FEV1, the mMRC score, and the BODE index, thus supporting the possible utility of the NLR as a clinically relevant tool for evaluating COPD progression and associated functional impairment [30]. Recent evidence supports the utility of the NLR as a systemic inflammatory marker in both stable COPD and during acute exacerbations (AECOPD). Gunay et al. [31] reported an elevated NLR in patients with an acute COPD exacerbation compared to those in the stable phase and healthy controls. These results support the hypothesis that the NLR may reflect an amplified systemic inflammatory response during AECOPD. Similarly, in the study by Furutate et al. NLR was associated with FEV_1_ and the BODE index (which integrates BMI, airflow obstruction, dyspnea and exercise tolerance), as well as other clinical parameters, such as nutritional status and the 6-MWD test. Notably, these associations remained statistically significant after adjusting for potential confounders, including age, sex, comorbidity burden (the Charlson index) and smoking status [32]. Consequently, the NLR may serve not only as a marker of disease severity, but also as a clinically relevant parameter for identifying and monitoring exacerbations in COPD patients [33].

In our study, IL-6 was correlated significantly with cigarette smoking history expressed in pack-years (r = −0.253, *p* = 0.024), and IL-8 was associated with biomass fuel exposure duration (r = 0.251, *p* = 0.045), underlining the contribution of both tobacco and environmental pollutants to the systemic inflammatory burden in COPD. Previous studies [34,35] have further emphasized the role of IL-6 as a central mediator of systemic inflammation in COPD, with elevated serum levels being significantly associated with reduced lung function, increased symptom severity, and a higher risk of acute exacerbations. Longitudinal data [36,37] have demonstrated that persistently elevated IL-6 levels are associated with excess mortality and decreased exercise tolerance, supporting its value as a prognostic biomarker in the management of COPD. These results draw special attention to the potential role of IL-6 not only as a marker of environmental and tobacco-related inflammatory response but also as a tool for risk evaluation and clinical decision-making in COPD.

Similarly, IL-8, a potent chemokine involved in neutrophil recruitment, plays a central part in COPD associated airway inflammation and tissue remodeling [38,39]. Elevated IL-8 has been linked to disease severity, airflow limitation, and the presence of chronic bronchitis [40]. IL-8 seems to be significantly increased in patients exposed to biomass smoke and other environmental pollutants, further supporting its role in pollutant-driven inflammatory responses [41]. Together, IL-6 and IL-8 reflect distinct but complementary aspects of COPD pathogenesis, with potential value as biomarkers for both disease monitoring and environmental risk assessment.

In COPD, cytotoxic T cells predominantly release TNF-alpha and IL-8, to the detriment of anti-inflammatory mediators (IL-10) [42,43]. This immune imbalance is then heightened by the activation of innate immune cells. A proinflammatory and prothrombotic phenotype is also observed in neutrophils, circulating monocytes and platelets, contributing to systemic inflammation and endothelial dysfunction. Higher concentrations of serum cytokines and acute-phase proteins are frequently reported in COPD patients, and some of these markers have shown correlations with disease severity. Nonetheless, the heterogeneity of baseline inflammatory markers reported in the literature reflects methodological variability, including differences in patient selection, the timing of blood collection and analytical protocols. These discrepancies underscore the need for standardized approaches in future studies assessing systemic inflammation in COPD [38,43,44].

Consistent with previous studies, our findings suggest that systemic inflammation in COPD surpasses the adaptive immune response, also involving innate immune components. Circulating monocytes, neutrophils, and platelets have been shown to exhibit both proinflammatory and prothrombotic profiles in patients with stable COPD. Proinflammatory cytokines and acute-phase proteins are elevated in COPD and correlate with clinical markers of disease severity [45,46]. Nevertheless, baseline levels of these inflammatory biomarkers vary substantially across studies, likely secondary to variations in patient selection, protocol, blood sampling schedule and analytical methodologies. These inconsistencies highlight the need for standardized protocols in future investigations.

Aghapour et al. mentioned the pivotal role of airway epithelial barrier dysfunction in the pathogenesis of chronic obstructive pulmonary disease, particularly in the context of sustained cigarette smoke exposure [47]. Their findings demonstrate that cigarette smoke impairs epithelial tight junctions, compromising the barrier’s integrity and allowing the penetration of environmental irritants and pathogens into the airway submucosa. This epithelial disruption contributes to a chronic proinflammatory state, not only at the local level but also through systemic “spill-over” mechanisms. These observations align with our findings and support the concept that epithelial dysfunction could act as a key link between chronic airway inflammation and systemic immune activation in COPD. Targeting epithelial barrier restoration could offer new therapeutic perspectives, aiming to improve both pulmonary and extrapulmonary outcomes. Moreover, assessing epithelial integrity might provide a valuable biomarker for disease severity and progression in future clinical research.

While IL-6 and IL-8 are well-established COPD inflammatory markers, our results suggest that IL-1 beta might also have a relevant function. As the difference in IL-1 beta levels were on the verge of conventional statistical significance (*p* = 0.053), this trend points toward its potential contribution to the systemic inflammatory burden in COPD. IL-1 beta is a key upstream mediator of innate immune activation, capable of amplifying inflammatory cascades and sustaining chronic airway inflammation. This subtle signal in our dataset highlights the need to look beyond the most studied cytokines and consider IL-1 beta as part of the broader inflammatory network that may influence disease trajectory. This perspective is supported by previous studies demonstrating significantly elevated IL-1 beta levels during acute exacerbations of COPD, with correlations to neutrophil percentage, C-reactive protein and smoking exposure. As such, IL-1 beta could be used as a sensitive marker of subclinical inflammation, even in stable phases of the disease. Incorporating IL-1 beta into future inflammatory profiling could provide deeper insights into individual risk for exacerbation and help refine biomarker-guided therapeutic approaches. These considerations are supported by the study by Zou et al. [48], who found significantly elevated IL-1 beta levels in patients with acute COPD exacerbations, in comparison with those with stable disease and healthy controls. Their study also found that IL-1 beta levels were positively correlated with serum CRP, neutrophil percentage and cumulative smoking exposure—markers that reflect systemic inflammatory stress. Furthermore, the strong association between IL-1 beta and TNF- alpha suggests a synergistic effect in driving neutrophilic inflammation. Taken together, these observations point to IL-1 beta as a meaningful indicator of inflammatory activity, not only during exacerbation but potentially even in stable phases of the disease. Recognizing and monitoring such signals may support earlier identification of patients at increased risk and help guide more proactive, individualized management strategies.

The 6 min walk test is a mortality marker in COPD and offers a practical way to understand how the disease affects routine physical activity. In our analysis, none of the analyzed inflammatory cytokines (IL-6, IL-8, IL-1 beta and TNF-alpha) showed significant correlations with the 6 MWD or its predicted percentage. Although several proinflammatory cytokines, such as IL-6, IL-8 and TNF-alpha, did not show statistically significant correlations with functional markers such as the 6 MWT or lung function in our sample, prior evidence supports their biological relevance. Their limited performance in this cohort may reflect sample size constraints, population heterogeneity or subthreshold systemic inflammation. Future longitudinal studies may better delineate their prognostic utility. Interestingly, among all markers analyzed, only the NLR demonstrated a statistically significant, albeit modest, correlation with the 6 MWD (r = 0.235, *p* = 0.039), which may reflect the broader role of immune cell imbalance in limiting physical performance.

Overall, our results highlight the complexity of the association between inflammation and functional status in COPD and underscore the need for multidimensional approaches when assessing disease impact and prognosis. Data from the SPACE study [49] indicate that patients with stable COPD who reported a high burden of 24 h respiratory symptoms also exhibited lower physical activity levels. This was associated with reduced performance in the 6 min walk test (6 MWT), underscoring the detrimental effect of persistent symptoms on functional capacity and daily activity. Liwsrisakun et al. [50] showed that decreased performance in the 6 MWT is independently associated with balance impairment in stable COPD. These findings reinforce the clinical value of the 6 MWT not only in evaluating functional capacity, but also as a practical tool for identifying individuals at increased risk of falls and guiding individualized rehabilitation strategies. Spruit et al. [51] reported that decreased aerobic capacity in COPD is influenced not only by airflow limitation, but also by other important factors, such as emphysema, depressive symptoms, and the subjective perception of dyspnea. Their findings underline the complexity of exercise intolerance in COPD, suggesting that reduced walking distance may occur even in patients with relatively preserved lung function.

Several previous studies [52,53,54] have evaluated systemic inflammatory biomarkers in COPD without adjusting for comorbidities, highlighting their potential as independent markers of disease severity and progression. In line with these findings, although comorbid conditions were not specifically addressed in our analysis, the biomarkers assessed reflect systemic processes that are increasingly recognized as central to COPD pathophysiology. Their associations with functional status support their clinical relevance regardless of other coexisting diseases. Given the high prevalence and impact of comorbidities in COPD, future studies should incorporate comorbidity stratification to enhance the interpretability and clinical utility of biomarker-based assessments.

### Limitations

This study has several limitations that should be acknowledged when interpreting the results. First, it was conducted in a single center, which may limit the generalizability of the findings to broader and more diverse populations. Although the prospective design allowed for systematic data collection and minimized recall bias, the relatively small sample size may have reduced the statistical power required to detect more subtle associations or differences between subgroups.

Second, while we adjusted for the main known confounding variables, the presence of residual confounding cannot be completely ruled out. Certain lifestyle and environmental factors were self-reported by participants, which may have introduced recall or reporting bias.

An additional factor contributing to the small sample size is that this study was conducted between January and December 2022, during the COVID-19 pandemic. During this period, many patients avoided or postponed medical visits due to public health restrictions, reduced access to healthcare facilities, or fear of infection. This situation may have introduced a selection bias, as patients who did present to the clinic might have differed in certain characteristics from those who did not seek medical care at that time.

Despite these limitations, the prospective nature of this study, the standardized methodology and the rigorous data collection process strengthen the internal validity of our findings. Future multicenter studies with larger and more diverse populations are warranted to validate our findings and to further investigate the prognostic role of NLR and other inflammatory biomarkers in COPD, as identified in our study.

## 5. Conclusions

The present study highlights the potential of the NLR as a promising and accessible inflammatory biomarker in COPD, with possible applications in assessing disease severity, identifying exacerbations and estimating prognosis. In our cohort, the NLR was associated with COPD severity and functional parameters, demonstrating good discriminatory capacity in identifying severe and very severe COPD (GOLD class 3–4). The NLR emerged as the most informative inflammatory biomarker, whereas no clinically relevant associations were observed for other markers, including IL-6, IL-8, IL-1β, TNF-α, ESR, hs-CRP, fibrinogen, PLR and LHR. Further studies are needed to validate these findings and to confirm the clinical utility of NLR in the diagnosis and follow-up of COPD.

## Figures and Tables

**Table 1:** Clinical characteristics across the 3 groups.

Clinical Characteristics	Total	GOLD 1–2	GOLD 3	GOLD 4	*p* Value
Age (years)	67.02 ± 9.303	66.38 ± 10.676	68.45 ± 5.847	66.00 ± 10.514	0.812
Male sex, n (%)	68 (68.0%)	31 (56.4%)	26 (78.8%)	11 (91.7%)	**0.011**
Smoking status					
Nonsmoker, n (%)	26 (26.0%)	19 (34.5%)	7 (21.1%)	-	**0.026**
Former smoker, n (%)	49 (49.0%)	20 (36.4%)	19 (57.6%)	10 (83.3%)	
Smoker, n (%)	25 (25.0%)	16 (29.1%)	7 (21.2%)	2 (16.7%)	
Biomass fuel exposure, n (%)	81 (81.0%)	41 (74.5%)	29 (87.9%)	11 (91.7%)	0.184
Duration of exposure of biomass fuel					
≤10 years	5 (6.2%)	3 (7.3%)	1 (3.4%)	1 (9.1%)	0.956
11–20 years	27 (33.3%)	15 (36.6%)	9 (31.0%)	3 (27.3%)	
21–40 years	47 (58.0%)	22 (53.7%)	18 (62.1%)	7 (63.6%)	
>40 years	2 (2.5%)	1 (2.4%)	1 (3.4%)	-	
**Clinical Parameters**					
BMI (kg/m^2^)	30.63 ± 6.842	30.82 ± 6.035	32.54 ± 7.543	24.48 ± 4.959	**0.003**
WBC (×10^9^/L)	7.45 ± 2.438	7.04 ± 2.012	8.10 ± 3.166	7.55 ± 1.521	0.313
LYM (×10^9^/L)	2.27 ± 2.053	2.35 ± 2.434	2.22 ± 1.707	1.99 ± 0.474	0.690
NEU (×10^9^/L)	5.10 ± 6.354	4.20 ± 1.924	6.71 ± 10.674	4.85 ± 1.364	0.063
PLT (×10^9^)	238.44 ± 76.417	240.40 ± 69.039	221.46 ± 89.314	276.17 ± 59.277	0.094
IL-8 (pg/mL)	148.02 ± 143.419	147.98 ± 140.369	153.74 ± 154.411	135.69 ± 143.932	0.922
TNF-alpha	16.84 ± 2.084	16.70 ± 0.985	17.25 ± 3.541	16.54 ± 0.790	0.787
IL-1 beta	35.18 ± 88.342	51.91 ± 112.587	15.67 ± 35.481	9.31 ± 8.649	**0.053**
IL-6	52.62 ± 54.363	61.89 ± 68.851	40.81 ± 24.166	40.43 ± 16.153	0.392
NLR	2.84 ± 4.514	2.24 ± 1.502	3.94 ± 7.551	2.60 ± 0.984	**0.015**
PLR	129.92 ± 66.879	128.11 ± 61.431	127.48 ± 82.248	144.92 ± 42.082	0.301
LHR	0.042 ± 0.0418	0.042 ± 0.0501	0.043 ± 0.0331	0.032 ± 0.0081	0.648
Nocturnal mean oxygen saturation, %		91.43 ± 3.489	91.16 ± 2.907	89.20 ± 0.707	0.286
Lowest nocturnal oxygen saturation, %		84.90 ± 6.860	83.80 ± 6.500	80.00 ± 1.414	0.296
Oxygen desaturation index /h					
Nocturnal heart rate, beats		76.88 ± 8.740	75.63 ± 10.169	66.92 ± 8.586	**0.005**

Continuous variables are expressed as mean values accompanied by standard deviation (SD), while categorical data are presented as absolute and relative frequencies (%). Abbreviations: BMI, body mass index; WBC, white blood cells; LYM, lymphocytes; NEU, neutrophils; PLT, platelets; IL-8, interleukin-8; TNF-alpha, tumor necrosis factor-alpha; IL-6, interleukin-6; IL-1 beta, interleukin-1 beta; NLR, neutrophil-to-lymphocyte ratio (normal range: 0.43–2.75 in males and 0.37–2.87 in females); LHR, lymphocyte-to-high-density lipoprotein ratio; PLR, platelet-to-lymphocyte ratio (normal range: 36.63–149.13 in males and 43.36–172.68 in females).

**Table 2:** Correlations between 6 MWT parameters and inflammatory biomarkers.

	6 MWD (m)		6 MWD % Pred	
	r (95% CI)	*p*	r (95% CI)	*p*
IL-6	0.194 (−0.051 ÷ 0.416)	0.119	0.142 (−0.104 ÷ 0.371)	0.256
IL-8	−0.011 (−0.252 ÷ 0.232)	0.932	0.009 (−0.234 ÷ 0.250)	0.943
IL-1 beta	−0.021 (−0.262 ÷ 0.222)	0.865	0.024 (−0.219 ÷ 0.264)	0.849
TNF-alpha	−0.029 (−0.269 ÷ 0.214)	0.815	−0.068 (−0.305 ÷ 0.177)	0.590
ESR	−0.078 (−0.296 ÷ 0.147)	0.496	−0.005 (−0.227 ÷ 0.218)	0.964
hs-CRP	−0.087 (−0.304 ÷ 0.138)	0.448	−0.011 (−0.233 ÷ 0.212)	0.921
Fibrinogen	−0.020 (−0.384 ÷ 0.349)	0.917	0.150 (−0.229 ÷ 0.489)	0.438
NLR	−0.235 (−0.435 ÷ −0.013)	0.039 *	−0.205 (−0.409 ÷ 0.019)	0.072
PLR	0.052 (−0.173 ÷ 0.271)	0.652	0.034 (−0.190 ÷ 0.255)	0.765
LHR	0.043 (−0.181 ÷ 0.263)	0.706	0.003 (−0.220 ÷ 0.225)	0.981

IL-8, interleukin-8; IL-6, interleukin-6; TNF-alpha, tumor necrosis factor-alpha; IL-1 beta, interleukin-1 beta; hs-CRP, high-sensitivity C-reactive protein; NLR, neutrophil-to-lymphocyte ratio; LHR, lymphocyte-to-high-density lipoprotein ratio; PLR, platelet-to-lymphocyte ratio; ESR, erythrocyte sedimentation rate. * statistically significant (*p* < 0.05).

**Table 3:** ROC analysis for NLR, PLR, LHR.

	AUC	*p*	Asymptotic 95% Confidence Interval		Cutoff Value		Sensibility	Specificity
			Lower Bound	Upper Bound				
**NLR**	**0.669**	**0.004**	**0.560**	**0.778**	2.305	62.2%	70.9%	
PLR	0.536	0.535	0.421	0.651	163.123	28.9%	85.5%	
LHR	0.459	0.486	0.343	0.576	0.0472	26.7%	85.5%	

AUC: area under the curve; LHR: lymphocyte-to-high-density lipoprotein ratio; NLR: neutrophil-to-lymphocyte ratio; PLR: platelet-to-lymphocyte ratio; ROC: receiver operating characteristic.

**Table 4:** Correlations between inflammatory biomarkers and duration of exposure of biomass fuel or pack-years.

Parameters	Duration of Exposure of Biomass Fuel		Pack-Years (Cigarette Smoking)	
	r (95% CI)	*p*	r (95% CI)	*p*
IL-6	−0.129 (−0.363 ÷ 0.121)	0.311	−0.253 (−0.448 ÷ −0.035)	0.024 *
IL-8	0.251 (0.006 ÷ 0.468)	0.045 *	0.167 (−0.055 ÷ 0.373)	0.139
IL-1 beta	0.002 (−0.244 ÷ 0.248)	0.988	−0.148 (−0.356 ÷ 0.074)	0.191
TNF-alpha	−0.081 (−0.320 ÷ 0.169)	0.526	−0.048 (−0.265 ÷ 0.173)	0.670
ESR	0.130 (−0.091 ÷ 0.339)	0.248	−0.045 (−0.239 ÷ 0.153)	0.659
hs-CRP	0.196 (−0.024 ÷ 0.397)	0.080	−0.036 (−0.231 ÷ 0.161)	0.720
Fibrinogen	0.011 (−0.371 ÷ 0.389)	0.958	0.150 (−0.198 ÷ 0.465)	0.397
NRL	−0.131 (−0.339 ÷ 0.090)	0.244	0.083 (−0.115 ÷ 0.275)	0.410
PLR	−0.120 (−0.329 ÷ 0.101)	0.288	−0.005 (−0.201 ÷ 0.192)	0.964
LHR	0.094 (−0.127 ÷ 0.306)	0.403	−0.042 (−0.237 ÷ 0.156)	0.677

IL-6, interleukin-6; IL-8, interleukin-8; IL-1 beta, interleukin-1 beta; NLR, neutrophil-to-lymphocyte ratio; PLR, platelet-to-lymphocyte ratio; LHR, lymphocyte-to-high-density lipoprotein ratio; ESR, erythrocyte sedimentation rate; TNF-alpha, tumor necrosis factor-alpha; hs-CRP, high-sensitivity C-reactive protein. * statistically significant (*p* < 0.05).

**Table 5:** Correlations between functional respiratory parameters and inflammatory biomarkers.

Parameter	FEV1%		FVC%		FEV/FVC Ratio	
	r (95% CI)	*p*	r (95% CI)	*p*	r (95% CI)	*p*
IL-6	0.205 (−0.015 ÷ 0.406)	0.068	0.238 (0.020 ÷ 0.435	0.033 *	0.166 (−0.056 ÷ 0.372)	0.141
IL-8	0.030 (−0.191 ÷ 0.248)	0.789	0.012 (−0.209 ÷ 0.231)	0.918	−0.043 (−0.261 ÷ 0.178)	0.702
IL-1 beta	0.180 (−0.041 ÷ 0.385)	0.109	0.172 (−0.049 ÷ 0.378)	0.127	0.184 (−0.037 ÷ 0.388)	0.102
TNF-alpha	−0.007 (−0.227 ÷ 0.213)	0.948	−0.054 (−0.270 ÷ 0.168)	0.636	0.010 (−0.210 ÷ 0.229)	0.929
ESR	0.120 (−0.078 ÷ 0.309)	0.233	0.102 (−0.096 ÷ 0.293)	0.311	0.046 (−0.152 ÷ 0.240)	0.652
hs-CRP	−0.022 (−0.218 ÷ 0.175)	0.828	0.039 (−0.159 ÷ 0.234)	0.700	0.008 (−0.189 ÷ 0.204)	0.940
Fibrinogen	0.073 (−0.272 ÷ 0.401)	0.682	0.211 (−0.137 ÷ 0.512)	0.231	0.078 (−0.267 ÷ 0.406)	0.661
NLR	−0.157 (−0.342 ÷ 0.041)	0.120	−0.053 (−0.247 ÷ 0.145)	0.599	−0.199 (−0.380 ÷ −0.002)	0.048 *
PLR	−0.027 (−0.222 ÷ 0.171)	0.793	0.041 (−0.157 ÷ 0.235)	0.686	0.000 (−0.196 ÷ 0.197)	0.999
LHR	0.038 (−0.159 ÷ 0.233)	0.705	0.040 (−0.157 ÷ 0.235)	0.689	0.098 (−0.101 ÷ 0.289)	0.333

IL-8, interleukin-8; IL-6, interleukin-6; TNF-alpha, tumor necrosis factor-alpha; IL-1 beta, interleukin-1 beta; NLR, neutrophil-to-lymphocyte ratio; LHR, lymphocyte-to-high-density lipoprotein ratio; PLR, platelet-to-lymphocyte ratio; ESR, erythrocyte sedimentation rate; hs-CRP, high-sensitivity C-reactive protein; FVC, forced vital capacity; FEV1, forced expiratory volume in the first second; * statistically significant (*p* < 0.05).

**Table 6:** Multivariate analysis—changes in FEV1 in relation to inflammatory markers.

Model	Unstandardized Coefficients	Standardized Coefficients	*p*	95.0% Confidence Interval for B	
	B	Beta		Lower Bound	Upper Bound
**(Constant)**	57.987		0.593	166.364	282.339
NLR	2.148	0.186	0.487	4.226	8.523
PLR	0.117	0.281	0.280	0.339	0.104
hs-CRP	1.691	0.164	0.527	7.214	3.832
ESR	0.027	0.015	0.958	1.051	1.105
Fibrinogen	0.004	0.024	0.930	0.093	0.101
IL-6	0.152	0.250	0.429	0.244	0.548
IL-8	0.034	0.266	0.339	0.108	0.039
TNF-alpha	0.031	0.001	0.996	12.124	12.186

IL-8, interleukin-8; IL-6, interleukin-6; TNF-alpha, tumor necrosis factor-alpha; NLR, neutrophil-to-lymphocyte ratio; ESR, erythrocyte sedimentation rate; PLR, platelet-to-lymphocyte ratio; hs-CRP, high-sensitivity C-reactive protein.

**Table 7:** Correlations between inflammatory markers and respiratory functional parameters.

	Lowest Nocturnal Oxygen Saturation (%)	Oxygen Desaturation Index/h		
	r (95% CI)	*p*	r (95% CI)	*p*
IL-6	0.144 (−0.110 ÷ 0.380)	0.264	0.001 (−0.249 ÷ 0.251)	0.994
IL-8	0.133 (−0.121 ÷ 0.370)	0.304	0.052 (−0.201 ÷ 0.298)	0.689
IL-1 beta	−0.102 (−0.343 ÷ 0.152)	0.432	−0.045 (−0.291 ÷ 0.207)	0.728
TNF-alpha	0.138 (−0.116 ÷ 0.375)	0.284	−0.099 (−0.340 ÷ 0.155)	0.444
ESR	−0.076 (−0.296 ÷ 0.152)	0.515	0.246 (0.022 ÷ 0.447)	0.032 *
hs-CRP	0.036 (−0.191 ÷ 0.259)	0.759	0.044 (−0.183 ÷ 0.267)	0.704
Fibrinogen	0.098 (−0.309 ÷ 0.475)	0.642	−0.043 (−0.430 ÷ 0.359)	0.840
NLR	−0.225 (−0.429 ÷ 0.000)	**0.051**	−0.066 (−0.287 ÷ 0.162)	0.573
PLR	−0.017 (−0.242 ÷ 0.209)	0.882	−0.023 (−0.247 ÷ 0.203)	0.842
LHR	−0.103 (−0.321 ÷ 0.126)	0.377	0.042 (−0.185 ÷ 0.265)	0.718

IL-8, interleukin-8; TNF-alpha, tumor necrosis factor-alpha; IL-6, interleukin-6; IL-1 beta, interleukin-1 beta; NLR, neutrophil-to-lymphocyte ratio (normal range: 0.43–2.75 in males and 0.37–2.87 in females); LHR, lymphocyte-to-high-density lipoprotein ratio; PLR, platelet-to-lymphocyte ratio (normal range: 36.63–149.13 in males and 43.36–172.68 in females); ESR, erythrocyte sedimentation rate; hs-CRP, high-sensitivity C-reactive protein; * statistically significant (*p* < 0.05).

## Data Availability

The data presented in this study are available on request from the corresponding author.
